# Supplementary material for: Harnessing the Power of Stem Cell Models to Study Shared Genetic Variants in Congenital Heart Diseases and Neurodevelopmental Disorders
Source: Cells. 2022 Jan 28;11(3):460. doi: 10.3390/cells11030460 (PMC8833927; doi:10.3390/cells11030460)
Supplement: Supplementary file 1 [file cells-11-00460-s001.zip › Cells_rev_Table_1.pdf]

Table S1: Summary of mouse models with tissue specific changes of *CTNNB1* expression. The floxed allele is first developed from Brault et al[21] with exon 2-6 deletion to fully knockout *CTNNB1.* The Flox(ex3) allele is first developed from Harada et al[48] with exon 3 deletion to stabilize β-catenin and mimic persistent canonical Wnt signaling activation. The Dm allele is first developed from Valenta et al[31] with both N- and C- terminal deletions to abolish transcriptional output by β-catenin but preserves its role in cell adhesion.

	Where	Phenotypes (Brain & Heart)	Reference
CTNNB1$^{floxed/floxed}$ or CTNNB1$^{dm/floxed}$ *Wnt1-Cre*	Brain, Spinal cord, Neural crest	Lack of midbrain, cerebellum and choroid plexus, underdeveloped forebrain; Cardiac outflow tract abnormalities	[21,30,31]
CTNNB1 overexpression *Nestin-enhancer driven expression of a truncated form with degradation site deleted*	Central nervous system progenitor cells	Macrocephaly	[32]
CTNNB1$^{floxed/floxed}$ *Foxg1-Cre*	Forebrain	Substantial reduction of forebrain structure	[33]
CTNNB1$^{floxed/floxed}$ *PV-Cre*	Parvalbumin interneurons	Impaired object recognition and social interactions; elevated repetitive behaviors; enhanced spatial memory	[34]
CTNNB1$^{floxed/floxed}$ *CAMKIIA-Cre*	Cortical excitatory neurons	Depression-like behavior, severe intellectual disability, increased anxiety, no autistic behavior	[35,36]
CTNNB1$^{+/flox(ex3)}$ *CAMKIIA-Cre*	Cortical excitatory neurons	Decreased social interest, increased repetitive behaviors	[37]
CTNNB1 missense *Thr653Lys in one allele WT in another allele*	Whole body	Brain abnormalities, behavioral abnormalities related with intellectual disability	[38]
CTNNB1$^{floxed/floxed}$ *Mesp1-Cre*	Early mesoderm progenitors	No cardiac looping, no right ventricle formation	[39]
CTNNB1$^{floxed/floxed}$ *ISL1-Cre*	Second heart field progenitors	No right ventricle formation	[40]
CTNNB1$^{-/floxed}$ *ISL1-Cre*	Second heart field progenitors	Dilated outflow tracts, smaller right ventricles, and thin-walled myocardium	[41]
CTNNB1$^{+/flox(ex3)}$ *ISL1-Cre*	Second heart field progenitors	Enlarged right ventricle	[40]
CTNNB1$^{floxed/floxed}$ *Mef2c-Cre*	Second heart field progenitors	Reduced size of right ventricle and defective distal outflow tract separation	[42]
CTNNB1$^{+/flox(ex3)}$ *Mef2c-Cre*	Second heart field progenitors	Dilated outflow tract, no distinct right ventricular structure	[42,43]
CTNNB1$^{floxed/floxed}$ *Nkx2.5-Cre*	Differentiated heart field cells	Reductions in ventricular size, especially right ventricle; a thinner compact layer in the ventricular wall	[40]
CTNNB1$^{dm/floxed}$	Cardiomyocytes	Abnormal tricuspid valve,	[44]

aMHC-Cre		hypoplastic right ventricle, malalignment of the interventricular septum, septal defects	
CTNNB1$^{+/floxed}$ *aMHC-Cre*	Cardiomyocytes	Smaller hearts with thin ventricular walls	45
CTNNB1$^{floxed/floxed}$ *My12-Cre*	Ventricular cardiomyocytes	Thinner compact layer of ventricular wall, reduced proliferation rate with compact and trabecular myocardial cells	46
CTNNB1$^{flox(ex3)/flox(ex3)}$ *My12-Cre*	Ventricular cardiomyocytes	No change of thickness of compact layer ventricular wall, increased proliferation rate with trabecular myocardial cells	46
CTNNB1$^{floxed/floxed}$ *Gata5-Cre*	Proepicardium/epicardium	Smaller heart, thinner compact myocardial layer, epicardial defects, no coronary vessels	47
