# Supplementary material for: Harnessing the Power of Stem Cell Models to Study Shared Genetic Variants in Congenital Heart Diseases and Neurodevelopmental Disorders
Source: Cells. 2022 Jan 28;11(3):460. doi: 10.3390/cells11030460 (PMC8833927; doi:10.3390/cells11030460)
Supplement: Supplementary file 1 [file cells-11-00460-s001.zip › Cells_rev_Table_2.pdf]

Table S2: Summary of shared CHD and NDD genes that are related with epigenetic regulation. Genes that are related with PRC complex are marked bold: KMT2A, KMT2C, KMT2D and ASH1L are trithorax group proteins that counteract with polycomb group proteins[91]. KDM5B affect binding of PRC group proteins in a subset of genes[69]. CHD7-bound regions are depleted of H3K27me3[92,93]. KDM6B removes H3K27me3 marks[94]. NSD1 has been shown to modulate PRC2 activity[95,96]. WHSC1 has been shown to alter binding of EZH2[97]. SETD5 occupancy correlates with H3K36me3 domains, but less with H3K27me3 domains[98].

**Group**	**Genes related with major histone modification that are shared by CHD and NDD**
H3-K4	Methyltransferase: **KMT2A** (brain[62], heart), **KMT2C** (brain[63,64], heart[65]), **KMT2D** (brain[66,67], heart[68])<br>Demethylase: **KDM5B** (brain[69,70], heart)<br>Methyl group reader: **CHD7** (brain[71], heart[72])
H3-K9	Methyl group reader: POGZ (brain[73,74], heart)<br>Acetyltransferase: KAT6A (brain, heart[75,76]), KAT6B (brain[76,77], heart)
H3-K27	Demethylase: **KDM6B** (brain[78], heart[79,80])<br>Acetyltransferase: EP300 (brain[81-83], heart[84])
H3-K36	Methyltransferase: **NSD1** (brain[76], heart), **WHSC1** (brain[85], heart[86]), **ASH1L** (brain[87,88], heart), **SETD5** (brain[89], heart[90])
