# Supplementary material for: Harnessing the Power of Stem Cell Models to Study Shared Genetic Variants in Congenital Heart Diseases and Neurodevelopmental Disorders
Source: Cells. 2022 Jan 28;11(3):460. doi: 10.3390/cells11030460 (PMC8833927; doi:10.3390/cells11030460)
Supplement: Supplementary file 1 [file cells-11-00460-s001.zip › Supplementary Material v5 XC.pdf]

Supplementary Methods:

GO analysis of CHD genes

CHD gene information is gathered from Jin et al.,2017. Total input CHD genes are 705 to the geneontology.org, using both biological process database and molecular function database. False discovery rate < 0.05 is thought to be significantly enriched GO term.

GO analysis of NDD genes

NDD gene information is gathered from Homsy et al.,2015. Risk genes are filtered (exclude pseudogenes, bacterial artificial chromosome clone, lncRNA and read-through variant). Total input NDD genes are 1153 to the geneontology.org, using both biological process database and molecular function database. False discovery rate < 0.05 is thought to be significantly enriched GO term.

Supplementary Tables:

Table 1: Total input CHD genes.

Table 2: Total input NDD genes

Table 3: GO terms of CHD genes (Biological Process dataset)

Table 4: GO terms of NDD genes (Biological Process dataset)

Table 5: GO terms of CHD genes (Molecular Function dataset)

Table 6: GO terms of NDD risk genes (Molecular Function dataset)
